# An Effective Optical Dual Gas Sensor for Simultaneous Detection of Oxygen and Ammonia

**DOI:** 10.3390/s19235124

**Published:** 2019-11-22

**Authors:** Sajal Biring, Annada Sankar Sadhu, Moumita Deb

**Affiliations:** Department of Electronic Engineering and Organic Electronics Research Center, Ming Chi University of Technology, New Taipei City 24301, Taiwan; annadamcut@gmail.com (A.S.S.); moumitadeb644@gmail.com (M.D.)

**Keywords:** optical dual sensor, oxygen, ammonia, ethyl cellulose, cellulose acetate

## Abstract

The development of a simple, low-cost sensor for the effective sensing of multiple gases in industrial or residential zones has been in high demand in recent days. In this article, we have proposed an optical sensor for the dual sensing of oxygen (O_2_) and ammonia (NH_3_) gases, which consists of oxygen and ammonia-sensitive fluorescent dyes coated individually on both sides of a glass substrate. An ethyl cellulose (EC) matrix doped with platinum (II) meso-tetrakis (pentafluorophenyl) porphyrin (PtTFPP) serves as the oxygen-sensing material, whereas the NH_3_-sensing material includes an eosin Y fluorescent indicator immobilized within a cellulose acetate (CA) matrix. Both the oxygen and ammonia-sensitive materials were excited by the same LED light source with a 405 nm peak wavelength, while the corresponding emissions were detected separately for the selective sensing of the gases under study. The dual gas sensor exhibits maximum sensitivities of around 60 and 20 for oxygen and ammonia gases, respectively. The high sensitivity and selectivity of the proposed optical dual sensor suggests the feasibility of the simultaneous sensing of oxygen and ammonia for practical applications.

## 1. Introduction

A large number of optical chemical sensors have been reported based on several spectroscopic methods including absorptiometry, reflectometry, fluorescence, infrared and Raman spectroscopies, interferometry, and surface plasmon resonance. Among them, the sensors using analyte-sensing fluorescence dyes usually show better sensitivity. Multiple parameters such as decay time, energy transfer, fluorescence quenching, polarization, etc. can be measured simultaneously using optical sensors to achieve specific advantages [1]. Numerous individual O_2_ and NH_3_ sensors are already reported based on the fluorescence quenching of various molecules in the presence of analyte gases [2]. However, an efficient dual sensor for O_2_ and NH_3_ gases based on fluorescence quenching is still lacking. 

Oxygen is a colorless and odorless gas, and it is vital for living life. Oxygen toxicity usually begins to occur at partial pressures >50 kPa causing convulsions and other health problems. In addition, detection of the oxygen level is of paramount importance in different fields, such as food packing, biomedical technology, environmental monitoring, etc. An optical gas sensor can determine oxygen concentration by monitoring the reduction in the fluorescence intensity of a dye molecule via its quenching by oxygen. Basically, the reported optical sensors employ a fluorescence indicator embedded in solid matrix, such as a polymer [2,3,4,5] or a sol–gel matrix [6,7,8,9] for hosting oxygen-sensitive fluorophores and assisting oxygen to penetrate into supporting matrices. Therefore, the properties of the oxygen-sensitive fluorescent dyes and the supporting matrices are important for the fluorescence-based optical sensors. Many studies have suggested ethyl cellulose (EC) as a matrix for detecting the O_2_ gas [2,3], since it shows high oxygen permeability, good mechanical and chemical stability, and excellent optical transparency in the visible light. The dye platinum (II) meso-tetrakis(pentafluorophenyl) porphyrin (PtTFPP) embedded in the EC matrix shows a high potential as a novel optical oxygen sensor material because of its good absorption in visible light, a strong fluorescence at room temperature, and a large Stokes shifts. 

On the other hand, ammonia gas is also a noxious and toxic gas. It irritates the respiratory system, skin, and eyes at low concentration of approximately 50 ppm (mg/L), while immediate and severe irritation occurs in the nose and throat at about 500 ppm, and induces pulmonary edema at about 1000 ppm [10]. The detection of accurate NH_3_ levels is critical in clinical samples, because the level is sensitive to the functions of many organs, such as kidney and liver [11,12]. There are multiple reasons behind the necessity of detecting ammonia in the fields of environmental monitoring, chemical industry, automotive applications, and medical diagnostics [13]. Previous investigations on NH_3_ sensing films made of matrices such as microparticles [14], silicon rubber [15], sol–gel [16], TiO_2_ thin film [17], and cellulose acetate (CA) [18] revealed analyte-dependent optical properties such as absorbance and luminescence. Most of these sensors have displayed improved sensitivity, stability, and reversibility. In this study, eosin Y and CA were chosen as the indicator and the supporting matrix, respectively. SiO_2_ nanoparticles (4 micron) were employed as network modifiers to increase the sensitivity of the ammonia sensor by taking advantage of the increased surface-to-volume ratio. CA has a high transmittance in visible light and permeability to water and ions. Interestingly, the hydrophobic eosin Y shows reasonable compatibility with CA.

A simple, low-cost optical dual sensor was utilized to detect O_2_ and NH_3_ gas based on the independent fluorescence quenching of the sensing materials. The proposed dual sensor was fabricated by coating the ammonia-sensing material on one side of a glass substrate and the oxygen-sensing material on the other side of that glass substrate. The two emission wavelengths sensitive to the selective gases were detected simultaneously. The newly developed sensing film is intended for medical, biological, and environmental applications. 

## 2. Materials and Methods

### 2.1. Materials 

The glass substrate (0.7 mm) was purchased from Corning (New York, NY, USA); platinum(II) meso-tetrakis (pentafluorophenyl) porphyrin (PtTFPP) was purchased from Frontier Scientific, (Logan, UT, USA); cellulose acetate (CA) powder was purchased from Showa Chemicals (Tokyo, Japan) and ethyl cellulose (EC) was purchased from Tokyo Chemical Industry Co., LTD (TCI) (Chuo City, Japan). Other reagents such as EtOH (99.5%) and SiO_2_ (4 micron, 99.9%) were purchased from ECHO Chemical co Ltd. (Miaoli, Taiwan). Eosin Y (99%) and acetic acid (99%) were purchased from Sigma Aldrich (St. Louis, MO, USA). Tetrahydrofuran (THF, 99.9%) and toluene (99.8%) were purchased from TEDIA (Fairfield, CT, USA). The received chemicals were used without any further purification.

### 2.2. Methods 

#### 2.2.1. Preparation of Oxygen and Ammonia Sensing Material 

Initially, a supporting matrix of the oxygen sensor was prepared by dissolving 1.25 gm of EC in 10 mL of toluene and 2.25 mL of EtOH (99.5%) as described elsewhere [3]. The solution was capped and stirred magnetically until it was turned into a transparent glue-like state. On the other hand, 0.05 gm of PtTFPP dye was dissolved into 10 mL of tetrahydrofuran (THF 99.9%) to produce a highly homogeneous solution for improving the oxygen sensitivity [4]. Finally, the oxygen-sensitive material was prepared by mixing the 100-µL EC matrix and 20-µL PtTFPP/THF solutions. 

The supporting matrix of the ammonia sensor was formulated by dissolving 0.22 gm of CA powder in 10 mL of acetic acid under stirring at 40 °C to form a transparent solution [18]. The ammonia-sensing indicator solution was prepared by dissolving 0.05 gm of eosin Y dye in 10 mL of THF. Finally, the ammonia-sensitive material was prepared by mixing the 50-µL CA matrix and 100-µL eosin Y/THF solutions with 1 mg of silica nanoparticles, which was then stirred mechanically at room temperature.

**Figure 1:**
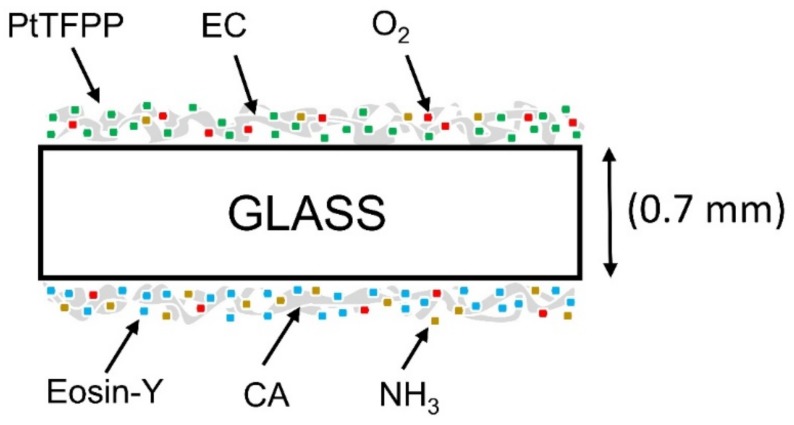
Schematic structure of the NH_3_–O_2_ dual gas sensor.

The oxygen-sensing material was spin coated (150 rpm for 20 sec) on one surface of a 0.7 mm glass substrate, and the ammonia-sensing material was spin coated (150 rpm for 20 sec) on the other surface of the glass substrate. The residual solvent in the sample was evaporated off at room temperature for 24 h to obtain the proposed dual gas sensor for O_2_ and NH_3_, as shown schematically in Figure 1.

#### 2.2.2. Instrumentation

**Figure 2:**
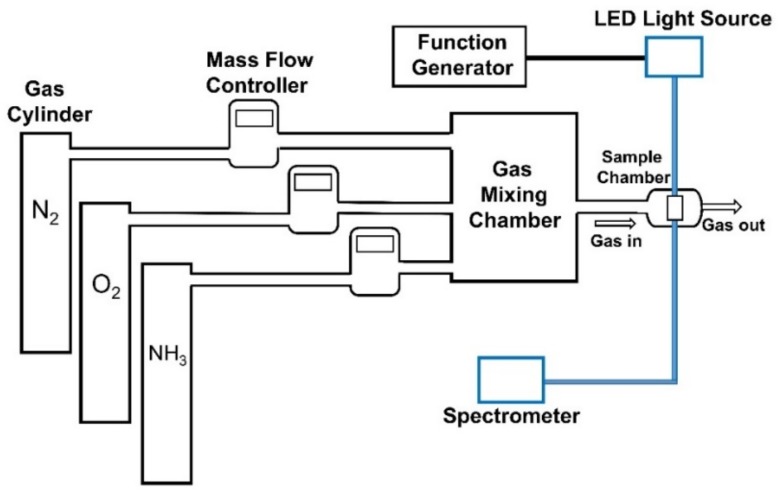
Experimental setup for optical dual sensor system.

Figure 2 illustrates the schematic diagram of the experimental setup used to characterize the performance of the optical dual sensor. In this experiment, the fluorescence excitation was provided by an LED with a central wavelength of 405 nm driven by an arbitrary waveform generator (TGA1240, Thurlby Thandar Instruments (TTi) Ltd., Huntington, UK) at 10-kHz frequency. A USB4000 fiber optic spectrometer (U.S. Ocean Optics, Inc., Largo, FL, USA) was used for measurement of the emission spectra, i.e., the relative fluorescence intensity. The concentration of O_2_ and NH_3_ were adjusted by mixing O_2_, NH_3_, and N_2_ in a mixing chamber through mass flow controllers (Aalborg instruments and Controls Inc., New York, NY, USA, Model GFC 17) at room temperature. A UV-Visible Spectrophotometer was used to measure the absorption spectra of dual-sensing materials. A spin coater (SWIENCO, Taiwan) was used to prepare the thin films of the sensing materials on a glass substrate.

## 3. Basic Theory

The quenching of a fluorophore depends on several factors. In the simplest scenario of a fluorophore in a homogeneous microenvironment, quenching follows the Stern–Volmer (S-V) equation [19], i.e.,

I_0_/I = 1 + K_sv_[Q] (1)

where I_0_ and I represent the steady-state fluorescence intensities in the absence and presence of a quencher; K_sv_ is the Stern–Volmer quenching constant; and [Q] is the concentration of quencher molecules or analyte molecules in this case. In the ideal case, the plot of I_0_/I versus [Q] is linear to the slope of K_sv_. The linearity of this equation describes the behavior of dynamic quenching. The above equation is required to be modified for the real case where not all the molecules are sensitive to the quencher, and the modified S–V equation can be written as [19,20]:

(2) $$\;\;\;\frac{I_{0}}{I}={\left(\frac{f}{1+K_{SV}\left[Q\right]}+\left(1-f\right)\right)}^{-1}$$

where I_0_ and I represent the steady-state fluorescence intensities in the absence and presence of the quencher molecules, respectively; K_sv_ and [Q] are the Stern–Volmer quenching constant and the concentration of quencher molecules respectively; and f is the fraction of the fluorescence caused by the sensitive molecules in a quencher-free environment. 

## 4. Results and Discussion

### 4.1. Optical Characterizations of the Sensing Materials

**Figure 3:**
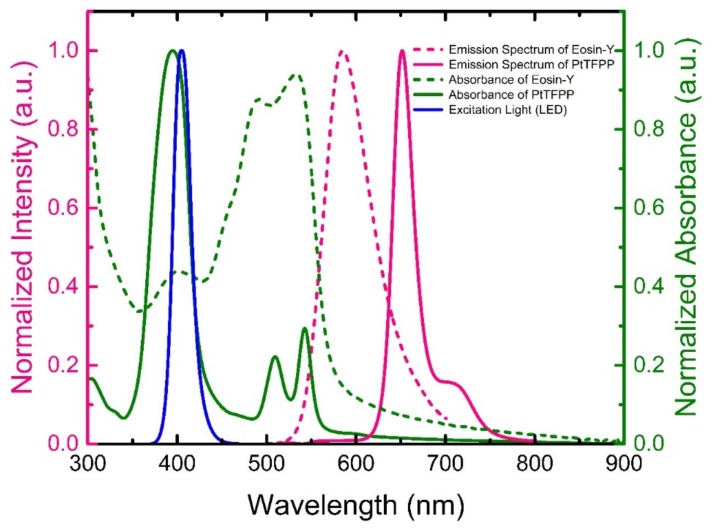
Absorption (green-colored) and fluorescence (pink-colored) spectra of the sensing materials used in the optical dual sensor. Absorption and fluorescence spectra were captured from individual materials. The blue curve represents the spectrum of excitation light source (LED).

Figure 3 depicts the absorption and fluorescence spectra of the materials (PtTFPP and eosin Y), respectively. The absorption spectra were captured from individual materials, while the fluorescence spectrum was captured from the dual sensor i.e., the combined fluorescence spectrum of PtTFPP and eosin Y materials. The central peaks of the absorption spectra for PtTFPP and eosin Y are 400 nm and 534 nm respectively. Besides, eosin Y shows a shoulder peak at around 400 nm. As a consequence, both the NH_3_ indicator, eosin Y, as well as the O_2_ indicator, PtTFPP, could be excited efficiently by a single 405-nm LED light source exhibiting strong fluorescence emissions at 582 nm and 650 nm, respectively. Thus, the individual detection of O_2_ and NH_3_ gases are performed without any ambiguity by monitoring the well-resolved bright emissions of sensing materials. The shoulder at ~720 nm in the fluorescence spectrum of the dual sensor belongs to the fluorescence spectrum of the PtTFPP material. 

### 4.2. O_2_ Sensing Properties of Optical Dual Sensor 

**Figure 4:**
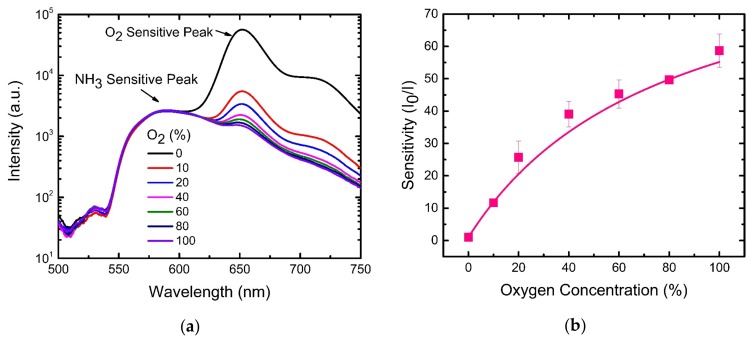
(**a**) Emission spectra (semi-log plot) of optical dual sensors at different oxygen concentrations (NH_3_-free environment) (**b**) Stern–Volmer plot for oxygen sensing.

Figure 4a depicts the relative fluorescence spectra of the optical dual sensor at different oxygen concentrations. The dual sensor was excited by a 405 nm LED, and the fluorescence spectra was acquired by a spectrometer equipped with a CCD. The emission spectra of PtTFPP and eosin Y showed no (or minor) spectral overlap or crosstalk. It is obvious from Figure 4a that the emission spectrum of PtTFPP with a peak wavelength of 650 nm is highly sensitive to the concentration of O_2_. Interestingly, the emission spectrum of eosin Y was unaffected by the change of O_2_ concentration facilitating the unambiguous detection of NH_3_ in the presence of O_2_.

The Stern–Volmer plot for oxygen quenching in Figure 4b shows the relationship between oxygen concentration and the ratio of fluorescence intensities in the absence (I_0_) and presence (I) of oxygen. The plot shows downward curvature with increasing concentration ruling off the application of Equation (1). On the other hand, the plot could be described well by Equation (2) considering the emission of sensitive fluorophores leading to the best fitting. The corresponding fractional contributions and Stern–Volmer quenching constants extracted from the fitting are f = 0.98 and K_sv_ = 1.23 ppm^−1^, respectively. The maximum sensitivity to oxygen gas molecules achieved by our dual gas sensor is 60 at 100% oxygen environment.

### 4.3. NH_3_ Sensing Properties of Optical Dual Sensor

**Figure 5:**
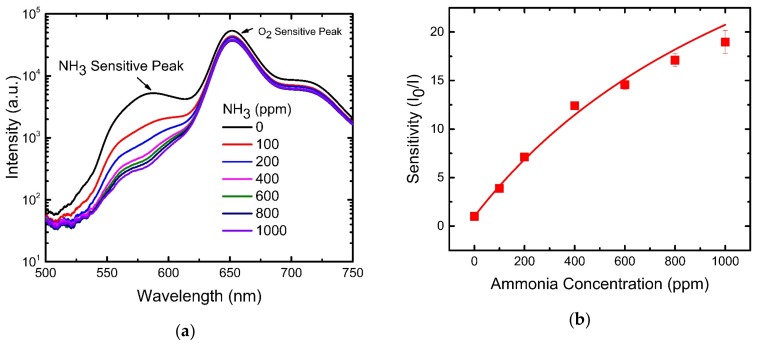
(**a**) Emission spectra (semi-log scale) from optical dual sensor at different ammonia concentrations (oxygen-free environment). (**b**) Stern–Volmer plot for ammonia sensing.

Figure 5a presents the emission spectrum of eosin Y and PtTFPP with peak wavelengths at 582 nm and 650 nm, respectively. The observed fluorescence intensities at 582 nm and 650 nm decrease with the increase in the NH_3_ concentration. The change in the emission spectrum from PtTFPP is almost negligible compared to the change in the fluorescence of eosin Y at 582 nm with an increasing concentration of NH_3_ from 0 ppm to 1000 ppm. 

The Stern–Volmer plot in Figure 5b also shows downward curvature, which is similar to the O_2_ sensing curve, and can also be described by Equation (2). The corresponding fractional contributions and Stern–Volmer quenching constants extracted from the best fitting are f = 0.98 and K_sv_ = 0.034 ppm^−1^, respectively. The maximum sensitivity to ammonia gas molecules achieved by our dual gas sensor is 20 in a 1000 ppm ammonia environment. 

### 4.4. Photostability of Optical Dual Sensor

**Figure 6:**
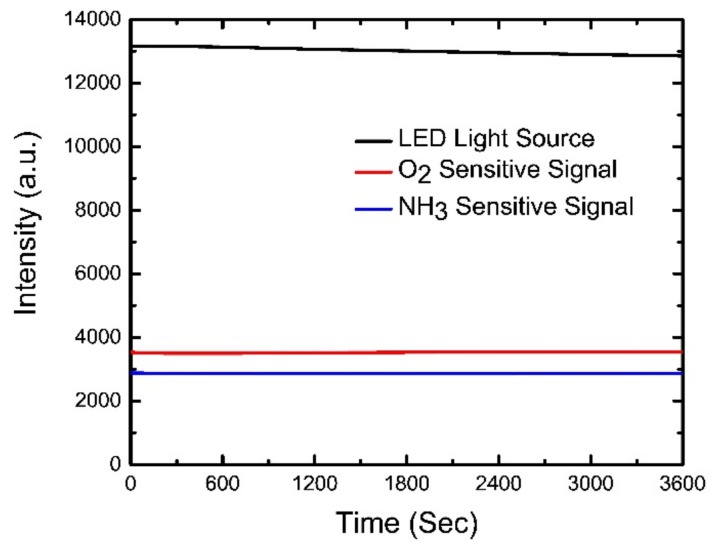
Photostability of optical dual sensor.

Photostability was tested by placing the optical dual sensor under a 405-nm wavelength pulse irradiated at room temperature in ambient light, and the change in fluorescence intensity was monitored. Figure 6 depicts that the relative fluorescence intensity of PtTFPP decreased by 0.05% and the relative fluorescence intensity of eosin Y decreased by 0.08% after continuous illumination for around 1 h in ambience. Neglecting the small changes under the experimental error, we may consider the optical dual sensor to be reasonably photostable over its operational time scale in the dual-sensor experiment. 

### 4.5. Response Time of Optical Dual Sensor

**Figure 7:**
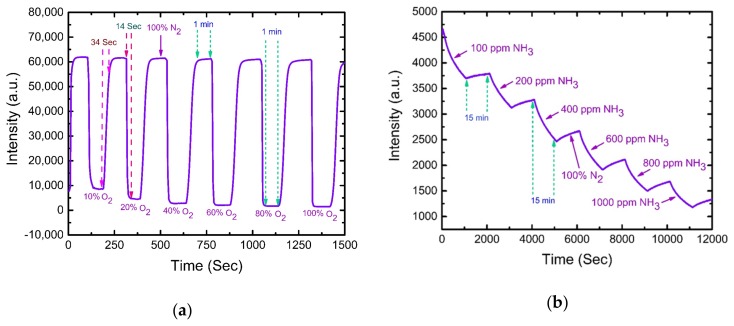
Dynamic response of dual sensor while switching alternatively from (**a**) 100% N_2_ to 100% O_2_ and (**b**) 100% N_2_ to 1000 ppm NH_3_ in gradual increasing steps of concentrations.

The rapid response of a sensor is necessary for practical applications. Figure 7a demonstrates the typical dynamic response of the optical dual sensor for O_2_ sensing while switching between 100% N_2_ to 100% O_2_ gases with gradual steps of increasing O_2_ concentrations. O_2_ gas was turned on for 1 min until the fluorescence intensity quenched to a saturated value, and then N_2_ gas was released to recover the fluorescence. The optical O_2_ sensor shows the response time of 14 s by switching from N_2_ to O_2_ and the recovery time of 34 s by switching from O_2_ to N_2_. Similarly, Figure 7b illustrates the dynamic response of the optical NH_3_ sensor. The concentration of NH_3_ gas was increased stepwise at every 15-min interval of the recovery of fluorescence intensity under 100% N_2_ atmosphere. The sensor exhibited a longer response (measured 22 min) and recovery time (hours) compared to the oxygen sensor. Fast recovery could be achieved by heating the NH_3_-sensitive material at 70 °C for 80 min. Thus, the proposed optical dual sensor provides a fast enough response for practical applications. 

### 4.6. Selectivity of Optical Dual Sensor

**Figure 8:**
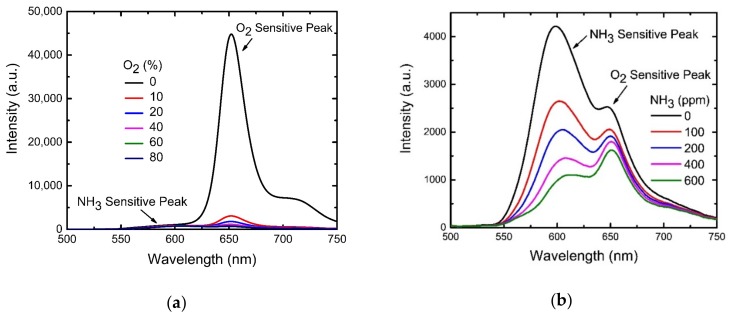
(**a**) O_2_-dependent emission spectra of optical dual sensor at 200 ppm NH_3_ concentration. Sensitivity plot at different oxygen concentrations for several fixed ammonia concentrations. (**b**) NH_3_-dependent emission spectra of optical dual sensor at 40% O_2_ concentration.

In most environmental and clinical situations, NH_3_ and O_2_ gases are present in a mixed form. Therefore, to estimate the concentration of individual gas components accurately, the dual sensor should not suffer from cross-sensitivity. To address the cross-sensitivity effect, we measured the spectral response of our proposed dual sensor at various mixtures of NH_3_ and O_2_ with different ratios. The spectral response (Figure 8a) of the dual sensor at different O_2_ concentrations in an ammonia environment fixed at 200 ppm is selectively sensitive to the O_2_ gas in the gas mixtures, i.e., the fluorescence of the ammonia-sensitive molecules is not affected with the change in O_2_ concentrations. On the other hand, the spectral response (Figure 8b) of the dual sensor at different NH_3_ concentrations in an environment of 40% O_2_ shows severe cross-sensitivity. It is obvious from the spectra that both the peaks that are individually responsible for NH_3_ and O_2_ sensing influence each other to some extent; in other words, we observe a spectral overlap or crosstalk in between the NH_3_ and O_2_-sensing signals. 

**Figure 9:**
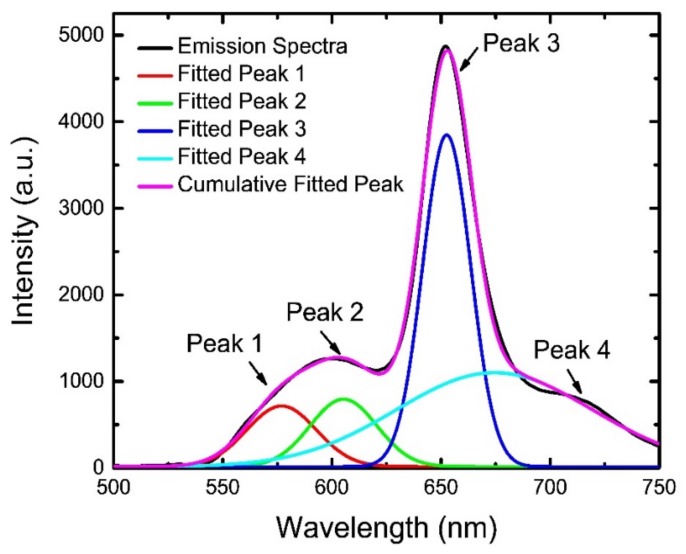
Gaussian fitting and decomposition of peaks for a spectrum acquired at 10% O_2_ and 100 ppm NH_3_ atmosphere.

To resolve this issue and to extract the data with better accuracy, we fitted the spectra with a Gaussian profile and extracted the intensity of the individual peak, as depicted in Figure 9 for an example. Figure 9 shows an emission spectrum (black line) of the dual sensor in an environment of 10% O_2_ and 100 ppm NH_3_. A Gaussian fitting (pink line) of this spectrum generates four individual peaks (red, light green, blue, and light blue lines) after decomposition, wherefrom we could estimate the peak intensities at 582 nm (peak 1) and at 650 nm (peak 3) for NH_3_ and O_2_ sensing respectively without any ambiguity. This method was employed to all the spectra for estimation of the corresponding peak intensity. 

**Figure 10:**
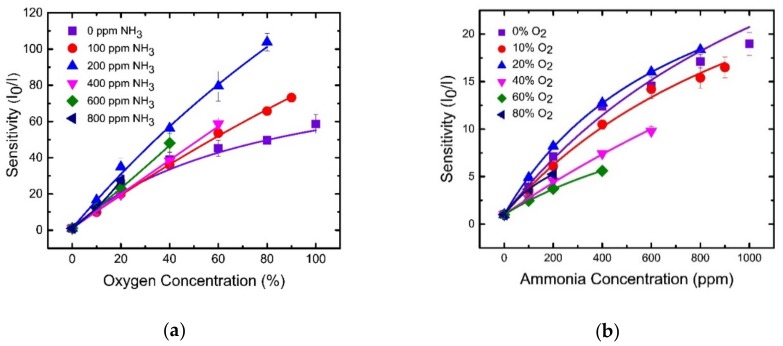
Cross-sensitivity data for O_2_ and NH_3_. (**a**) Sensitivity plot at different oxygen concentrations for several fixed ammonia concentrations. (**b**) Sensitivity plot at different ammonia concentrations for several fixed oxygen concentrations.

Finally, for practical applications, the calibration curves for a dual sensor influenced by the cross-sensitivity effect were plotted as presented in Figure 10. The figures reveal a negligible cross-sensitivity effect at lower O_2_ concentrations (below 20%) and NH_3_ concentrations (below 200 ppm), whereas they reveal non-negligible cross-sensitivity at higher concentrations of O_2_ and NH_3_ gases.

## 5. Conclusions

This work presents a novel optical sensor for the dual sensing of oxygen and ammonia concentrations based on sensing materials coated on a glass substrate. The optical dual sensor was fabricated using PtTFPP and eosin Y as the fluorescent indicators for oxygen and ammonia, respectively. It has been shown that the sensitivities of the oxygen and ammonia sensors are 60 and 20 respectively in an oxygen–ammonia mixed chamber. The response and recovery time for oxygen sensing are in seconds (14 s and 34 s, respectively), while these are in minutes (22 min) and hours respectively for ammonia sensing. The proposed dual gas sensor is highly reproducible and regenerative to sense the gases for multiple times. The optical dual sensor developed in this study enables the simultaneous sensing of oxygen and ammonia concentrations effectively and could find practical applications in various fields, including medical and industrial sections.

## References

[1] Wolfbeis O.S. (2005). Materials for fluorescence-based optical chemical sensors. J. Mater. Chem..

[2] Amao Y., Miyashita T., Okura I. (2001). Platinum tetrakis (pentafluorophenyl) porphyrin immobilized in polytrifluoroethylmethacrylate film as a photostable optical oxygen detection material. J. Fluor. Chem..

[3] Chu C.S., Syu J.J. (2017). Optical sensor for dual sensing of oxygen and carbon dioxide based on sensing films coated on filter paper. Appl. Opt..

[4] Yeh T.S., Chu C.S., Lo Y.L. (2006). Highly sensitive optical fiber oxygen sensor using Pt(II) complex embedded in sol-gel matrices. Sens. Actuators B Chem..

[5] Chu C.S., Lo Y.L. (2007). High-performance fiber-optic oxygen sensors based on fluorinated xerogels doped with Pt(II) complexes. Sens. Actuators B Chem..

[6] Basu B.J. (2007). Optical oxygen sensing based on luminescence quenching of platinum porphyrin dyes doped in ormosil coatings. Sens. Actuators B Chem..

[7] Chu C.S., Lo Y.L., Sung T.W. (2010). Enhanced oxygen sensing properties of Pt(II) complex and dye entrapped core-shell silica nanoparticles embedded in sol-gel matrix. Talanta.

[8] Chu C.S., Lo Y.L. (2011). Highly sensitive and linear calibration optical fiber oxygen sensor based on Pt(II) complex embedded in sol-gel matrix. Sens. Actuators B Chem..

[9] Elosua C., de Acha N., Hernaez M., Matias I.R., Arregui F.J. (2015). Layer-by-Layer assembly of a water-insoluble platinum complex for optical fiber oxygen sensors. Sens. Actuators B Chem..

[10] Timmer B., Olthuis W., Berg A.V.D. (2005). Ammonia sensors and their applications—A review. Sens. Actuators B Chem..

[11] Damink S.W.M.O., Deutz N.E.P., Dejong C.H.C., Soeter P.B., Jalan R. (2002). Interorgan ammonia metabolism in liver failure. Neurochem. Int..

[12] Cohen B.I. (2002). The significance of ammonia/gamma-aminobutyric acid (GABA) ratio for normality and liver disorders. Med. Hyphothess.

[13] Ryer-Powder J.E. (1991). Health effects of ammonia. Plant/Oper. Prog..

[14] Takagai Y., Nojiri Y., Takase T., Hinze W.L., Butsugan M., Igarashi S. (2010). “Turn-on” fluorescent polymeric microparticle sensors for the determination of ammonia and amines in the vapor state. Analyst.

[15] Vaughan A.A., Baron M.G., Narayanaswamy R. (1996). Optical ammonia sensing films based on an immobilized metalloporphyrin. Anal. Commun..

[16] Sung T.W., Lo Y.L. (2013). Ammonia vapor sensor based on CdSe/SiO_2_ core-shell nanoparticles embedded in sol-gel matrix. Sens. Actuators B Chem..

[17] Castillero P., Roales J., Lopes-Costa T., Sanchez-Valencia J.R., Barranco A., Gonzalez-Elip A.R., Pedrosa J.M. (2017). Optical gas sensing of ammonia and amines based on protonated porphyrin/TiO_2_ composite thin film. Sensors.

[18] Peng L.R., Yang X.H., Yuan L.B., Wang L.L., Zhao E.M., Tian F.J., Liu Y.X. (2011). Gaseous ammonia fluorescence probe based on cellulose acetate modified microstructured optical fiber. Opt. Commun..

[19] Lakowicz J.R. (2006). Principles of Fluorescence Spectroscopy.

[20] Carraway E.R., Demas J.N., DeGraff B.A. (1991). Luminescence quenching mechanism for microheterogeneous systems. Anal. Chem..

